# Chagas disease in the 21st Century: a public health success or an emerging threat?

**DOI:** 10.1051/parasite/2014012

**Published:** 2014-03-10

**Authors:** Kevin M. Bonney

**Affiliations:** 1 Department of Biological Sciences, Kingsborough Community College, City University of New York 2001 Oriental Boulevard Brooklyn, New York 11235-2398 USA

**Keywords:** Chagas disease, *Trypanosoma cruzi*, Neglected tropical disease

## Abstract

Chagas disease, caused by the protozoan parasite *Trypanosoma cruzi*, is a major public health burden in Latin America and a potentially serious emerging threat to a number of countries throughout the world. Although public health programs have significantly reduced the prevalence of Chagas disease in Latin America in recent decades, the number of infections in the United States and non-endemic countries in Europe and the Western Pacific Region continues to rise. Moreover, there is still no vaccine or highly effective cure available for the approximately 10 million people currently infected with *T. cruzi*, a third of which will develop potentially fatal cardiomyopathy and/or severe digestive tract disorders. As Chagas disease becomes an increasingly globalized public health issue in the twenty-first century, continued attentiveness from governmental and health organizations as well as improved diagnostic tools, expanded surveillance and increased research funding will be required to maintain existing public health successes and stymie the spread of the disease to new areas and populations.

## Abstract (fr)

La maladie de Chagas, causée par le protozoaire parasite *Trypanosoma cruzi*, est un problème de santé publique majeur en Amérique latine et une menace émergente potentiellement grave dans un certain nombre de pays à travers le monde. Bien que les programmes de santé publique aient considérablement réduit la prévalence de la maladie de Chagas en Amérique latine au cours des dernières décennies, le nombre d’infections aux États-Unis et les pays non-endémiques d’Europe et de la Région du Pacifique occidental continue d’augmenter. En outre, il n’existe encore aucun vaccin ou remède très efficace disponible pour les quelque 10 millions de personnes actuellement infectées par *T. cruzi*, dont un tiers va développer une cardiomyopathie potentiellement mortelle et / ou des troubles digestifs sévères. Comme la maladie de Chagas devient un problème de plus en plus globalisé de santé publique au XXIe siècle, une attention continue des organisations gouvernementales et de santé ainsi que des outils de diagnostic améliorés, une surveillance accrue et un financement accru de la recherche seront nécessaires pour maintenir les bons résultats actuels de santé publique et entraver la propagation de la maladie à de nouvelles régions et populations.

## Introduction

Chagas disease (Human American Trypanosomiasis) was first described in 1909 when Carlos Chagas identified the protozoan parasite *Trypanosoma cruzi* as the cause of an acute febrile illness afflicting Brazilian railroad workers [84]. It is likely that the insect vectors that spread *T. cruzi* had been transmitting the parasite among wild animals in Central and South America for millions of years before the disease crossed over into domestic animals and humans more than 9000 years ago [7, 26]. Approximately 200–300 years ago, as rapid conversion of the natural forest habitat of the vector into farmland created myriad opportunities for *T. cruzi* to spread to domesticated animals, Chagas disease became an endemic zoonosis [28]. Urbanization of rural populations in the mid-twentieth century, which involved the migration of large numbers of infected individuals to areas with a comparatively low risk of vectorial transmission, extended the endemic to cities. However, the disease has remained largely confined to poor rural areas. By the end of the twentieth century, Chagas disease had become widely recognized by the World Health Organization (WHO) and other public health authorities as a neglected tropical disease because it primarily affects low-income populations, is a major cause of chronic morbidity and mortality in developing tropical countries, and has been historically underrepresented in the allocation of health-promoting resources from research, governmental, and public aid organizations. Chagas disease is a serious public health burden in Latin America (Table 1), costing the region an estimated 662,000 disability-adjusted life-years (DALYs) of productivity as of 2008, nearly six times the socioeconomic toll of malaria (in terms of DALYs) in the region [48]. Because it disproportionately affects low-income individuals, who are least able to protect themselves against infection and seek and complete appropriate treatment, and has a substantially deleterious effect on the ability of those individuals to pursue education, earn income and save their earnings, Chagas disease is part of a self-propagating cycle of poverty in many endemic regions. More recently, widespread emigration of Latin Americans, including a large number who are infected with *T. cruzi,* has resulted in an emerging public health threat in historically non-endemic areas of the world such as the United States, Canada, Western Europe, Japan, and Australia (Table 1) [28, 36, 71]. The total economic toll attributed to the disease each year is estimated at over $7 billion USD, with more than 10% of this cost being incurred in the United States and Canada [36, 54].

**Table 1.** Estimated number of cases of *Trypanosoma cruzi* infection by country, as of 2009 [54, 55].

Region	1–999	1000–9999	10,000–99,999	100,000–999,999	>1,000,000
Central and South America			Belize Costa Rica French Guyana Guyana Nicaragua Panama Suriname Uruguay	Bolivia Chile Colombia Ecuador El Salvador Guatemala Honduras Paraguay Peru Venezuela	Argentina Brazil
North America		Canada		United States	Mexico
Europe	Austria Croatia Denmark Germany Greece Luxembourg Netherlands Norway Portugal Romania Sweden	Belgium France Italy Switzerland United Kingdom	Spain		
Asia and Oceania		Australia Japan			
Africa					

In Latin America, *T. cruzi* infection most often occurs via vectorial transmission by a type of reduviid bug called a triatomine or “kissing bug”. Triatomines are nocturnal feeders that may live in a variety of environments surrounding human dwellings, including cracks and holes in the walls, ceilings, and floors of substandard housing structures. After taking a blood meal, infected triatomines often excrete feces contaminated with *T. cruzi* onto their host; *T. cruzi* can enter the bite wound or a nearby mucosal surface such as the conjunctiva when the victim inadvertently rubs these parasites across their skin [40]. Other routes of transmission include congenital, transfusion of contaminated blood, transplantation of organs from infected donors, ingestion of contaminated food or drinks, and accidental exposure (e.g. laboratory accidents). Once in the bloodstream of a mammalian host, *T. cruzi* is able to infect a variety of cell types throughout the body and establish a chronic infection.

During the acute phase of *T. cruzi* infection parasitemia is often high enough that diagnosis can be made through the microscopic examination of blood for the blood-form (trypomastigote stage) of the parasite. During the chronic stage of Chagas disease, diagnosis can be made serologically using enzyme-linked immunosorbent assay, indirect hemagglutination, indirect immunofluorescence, or immunochromatography to test for the presence of *T. cruzi-*specific immunoglobulin G (IgG). It is recommended that at least two different types of serological tests are used to analyze each potentially infected individual or sample, as notable heterogeneity has been observed among the results obtained using different testing methods, and because there is considerable risk of obtaining false positive results with individual tests due to cross-reactivity of anti-*T. cruzi* antibodies with antigens of closely related species of trypanosomatids [19, 41, 70, 81]. Thoroughly purifying antigenic preparations prior to analysis and selecting tests with the greatest specificity available can reduce the risk of obtaining false positive results with serological tests. Another potential diagnostic tool for *T. cruzi* infection is polymerase chain reaction (PCR) to assess the presence of *T. cruzi* DNA. However, despite promising results for effective use of PCR in the diagnosis of *T. cruzi* in certain instances in which serological diagnosis may be especially limited, such as in neonates with low parasitemia and in HIV co-infected patients, a combination of serological methods remains the preferred method of diagnosis due to generally higher sensitivity and commercial availability, and lower heterogeneity [19, 31, 41, 70].

After an incubation period of 5–40 days, 10–30% of infected individuals will begin to exhibit non-specific symptoms of acute Chagas disease, including abdominal pain, anorexia, fever, lymphadenopathy, malaise, rash and localized swelling around the site of infection [63]. The mortality rate of acute Chagas disease is 5–10%, usually due to acute myocarditis or meningoencephalitis, with the majority of deaths occurring in young children [82]. However, the majority of *T. cruzi*-infected individuals become asymptomatic carriers of the parasite, often with low or undetectable parasitemia (though *T. cruzi*-specific antibodies and DNA may remain at detectable levels in the blood) [1, 77, 83]. After several decades in this indeterminate disease state, during which there are no clinically overt symptoms of organ damage or abnormal electrocardiographic results, approximately 30–40% of asymptomatic carriers will develop chronic Chagas disease characterized by dilated cardiomyopathy leading to congestive heart failure, and/or by development of gastrointestinal disorders, the two most prominent being megacolon and megaesophagus [71]. The etiology of Chagas disease pathogenesis is complex and not completely understood, and may involve a combination of cellular and neuronal damage directly mediated by live *T. cruzi*, as well as indirect damage caused by immune responses to the parasite and self-antigens exposed during infection [18, 61]. A lengthy period of parasite persistence appears to be necessary for the induction of Chagas pathogenesis [18, 61]. A number of factors, including the host and parasite genetics, infective dose, route of transmission, number of reinfections, and initial and late host immune response affect the onset, severity and presentation of symptoms [5, 8, 20, 27, 38, 63].

Only two drugs, benznidazole and nifurtimox, have been shown to be effective enough to warrant widespread use in Chagas disease treatment. Benznidazole functions, in part, by inducing the formation of free radicals and other metabolites which bind to the nuclear and mitochondrial DNA of *T. cruzi*, leading to lethal DNA strand breaks [69]. Nifurtimox also exploits the parasite’s vulnerability to oxygen radicals by inhibiting the function of an enzyme *T. cruzi* requires for detoxifying such compounds [34]. It is widely recommended that treatment with one of these drugs be administered immediately following confirmative diagnosis to those experiencing symptoms of acute Chagas disease. Current treatment protocols are effectively curative in approximately 60% of all acute Chagas cases, but the success rate is only 10–20% for symptomatic chronic Chagas disease, and it remains to be proven whether anti-trypanosomal chemotherapy provides any substantial benefit to patients experiencing the asymptomatic indeterminate state of the disease [4, 12, 15, 23, 74]. Compared with adults, benznidazole treatment of *T. cruzi*-infected children is considerably more effective and better tolerated [4, 15]. In children, the overall rate of curative therapy has been reported as 71.5% for acute cases of Chagas disease, with >90% cure rates reported for cases of congenital infection if treatment is given within the first year of life [3, 15]. The cure rate of recent chronic Chagas disease in children (0–14 years of age) has been reported as 57.6%; however, this represents a minority of all chronic Chagas patients, as the majority of chronic Chagas patients are 15–69 years of age [3, 42, 58]. It is important to note that anti-*T. cruzi* treatment efficacy is difficult to accurately assess due to the inherent uncertainty of determining whether and when viable parasites have been completely eliminated from an infected individual. Due to the lack of a reliable early indicator of curative therapy, treatment efficacy is evaluated by the conversion of a previously positive serological test to negative, with long-term follow-up testing for a period of 10–20 years following treatment [52], Consequences of the current inability of physicians to obtain and disseminate timely and accurate information about treatment efficacy to patients include reluctance of patients to complete lengthy treatment regimens and seek follow-up counseling, and impaired ability of physicians to adequately assess and inform patients of their long-term risk of cardiac pathology [52].

In addition to its limited efficacy, treatment with benznidazole or nifurtimox poses a substantial risk of serious side effects, including digestive intolerance, hepatitis, peripheral neuropathy, and rash, which have been observed in 30–50% of treated individuals [36]. Use of these drugs is contraindicated during pregnancy and in patients with advanced kidney or liver disease, and may be logistically or economically prohibitive to other patient populations because a lengthy 60–90 day treatment regimen is often required [36, 71]. Recent estimates place the cost of treatment for an infected individual, depending on the level of care provided, at $46–$7981 USD per year in Colombia and $3000–$14,580 USD per year in Mexico [86]; the per capita GDP in both of these countries is under $10,000 USD according to the International Monetary Fund. These drugs are also not widely available in all areas, and many infected individuals in endemic countries have limited access to health care facilities. Even in the United States these drugs are available only if obtained directly from the Centers for Disease Control (CDC) under an investigational protocol because the United States Food and Drug Administration (FDA) has not yet approved them for routine use in the country. For both of these drugs to be effective, treatment for at least 60 days is required, which compounds the burden of these health, economic, and logistical impediments [71].

## Progress against Chagas disease in endemic regions

By some estimates, the number of people infected by *T. cruzi* worldwide has been reduced by 50% or more within the past 25 years, from a peak of 15–30 million in 1990 [32, 33], to a current total of 8–10 million [89]. After reaching a peak in the 1980s, the number of annual deaths from Chagas disease is estimated to have dropped from 45,000 in 1990 to approximately 12,000 in recent years [32, 60]. Although variations and inherent uncertainties in epidemiological methodology may have resulted in certain studies greatly overestimating the actual prevalence of *T. cruzi* infection, especially in earlier years, it is clear that extensive public health initiatives in endemic countries have been effective at significantly reducing the rate and risk of new infections.

Control of the insect vector that transmits *T. cruzi* infection has historically been the primary focus of public health programs aimed at reducing the prevalence of Chagas disease. For the first several decades following the identification of *T. cruzi* as the causative agent of Chagas disease there was no specific treatment available for human infections and common methods for reducing infestation of domestic dwellings such as the use of kerosene, cyanide gas, or flame throwers were crude and potentially destructive [33]. By the 1940s, focus had shifted to the development of insecticides and improvement of housing structures to limit the persistence and spread of the vector. Because domestic animals, especially dogs, can serve as epidemiologically important reservoirs for the parasite, efforts to eradicate the vector were extended to domestic animal dwellings [44, 59]. By the 1990s, the success of vector eradication programs had become evident in a number of localities and nations, largely due to the success of several large-scale multi-national initiatives.

The most notable of these programs are the Southern Cone Initiative (launched in 1991), the Andean Pact Initiative (launched in 1997) and the Central America Initiative (launched in 1997). The main objectives of these programs were to reduce vectorial transmission by eliminating populations of domestic vectors, to increase screening of blood donors in order to prevent transmission via transfusion, and to expand maternal screening to decrease the incidence of congenital transmission and ensure appropriate treatment of potentially infected neonates. In many regards these programs have been resoundingly successful. By 1999, the Pan-American Health Organization (PAHO) had declared that *Triatoma infestans*, the primary domestic vector of *T. cruzi* in rural areas of South America, had been effectively eliminated from human dwellings in Brazil, Chile, Uruguay, and large portions of Argentina, Bolivia, and Paraguay [60, 71, 78]. Efforts to eliminate *Rhodnius prolixus*, widely considered to be the second most important vector for the transmission of Chagas disease, have been similarly effective in Guatemala, Honduras, and El Salvador, and preliminary results indicate progress is being made in additional areas [33]. Eradication of *R. prolixus* from all of Central America is now considered feasible in the relatively near future [33]. As a result of vector control, the estimated number of *T. cruzi-*infected individuals dropped substantially in all ten South American countries targeted by the Southern Cone and Andean Pact Initiatives between 1980 and 2005, as well as for most of the countries targeted by the Central America Initiative [71]. The population deemed at risk for contracting Chagas disease also dropped markedly in the countries targeted by these initiatives. For example, the percent of the Chilean population deemed at risk for contracting the infection dropped from 63% to 5% between 1980 and 2005 [71]; Venezuela experienced a decrease in infection risk from 72% to 18% during the same time period [71].

Following widely successful efforts to reduce vectorial transmission of *T. cruzi*, blood transfusion became the primary cause of infection in many areas [66]. Mandatory screening of blood products began in many Latin American countries in 1988 [30], and by 2005 100% screening coverage had been achieved in 12 of those countries, with two additional countries achieving 99% coverage [60]. In Brazil, the percent of blood donor candidates who have Chagas disease decreased tenfold in the twenty-five years following 1980, from 4% to 0.4% [33]. These efforts have likely resulted in the prevention of millions of new infections. However, it is notable that the lowest rate of blood donor screening reported by a Latin American country in the aforementioned study, 80%, was in Bolivia [60]. This is of particular importance because Bolivia also has the highest rate of *T. cruzi* infection in the world, as well as the highest rate of seroprevalence among tested donors [6, 60]. Subsequently, at least one study has indicated that Bolivian immigrants in Europe are more than twice as likely as other Latin American immigrants to be infected with *T. cruzi* [6].

Congenital transmission accounts for over 15,000 [67] cases of *T. cruzi* infection each year, mostly in endemic regions, justifying its selection as the third main target of the major Latin American anti-Chagas initiatives of the 1990s. Since the late 1990s, a number of Latin American countries, including Argentina, Uruguay, and Paraguay, have implemented policies to routinely screen all pregnant woman and infants serologically for indications of *T. cruzi* infection. Because the risk of congenital *T. cruzi* transmission may last for years after a potential mother initially contracts the infection, and due to widespread emigration of infected individuals, congenital transmission is of concern in both endemic and non-endemic areas, and in areas where vector transmission has been interrupted or eliminated. Due to the substantial side effects and unclear teratogenic risks of available trypanocidal medications and a lack of other therapeutic options, there is no reliable method for preventing congenital infection. The most effective strategy for limiting the spread of congenital Chagas disease is widespread dissemination of available treatment to *T. cruzi*-infected women of child-bearing age coupled with routine serological screening of pregnant mothers and prompt treatment of children born to infected mothers. Despite therapeutic limitations, extant deficiencies in the requisite diagnostic and clinical infrastructure, and low rates of coverage in impoverished rural areas, there is evidence that the rate of congenital transmission and the morbidity and mortality associated with congenital transmission is declining in a number of areas [78, 85].

The significant decrease in new infections, hospitalizations, loss of healthy years of life and fatalities from Chagas disease achieved by the efforts of successful public health campaigns has resulted in a substantial economic benefit to the world, and to Latin America in particular. Although the sum of this economic benefit is difficult to measure precisely, analysis conducted in the year 2000 estimated that the $420 million USD that the Brazilian government had invested in Chagas disease control between 1975 and 1995 had already resulted in over $3 billion USD in benefits, yielding a net return of $7.16 USD for every dollar invested [2, 33].

## The emerging threat of Chagas disease

While the prevalence of Chagas disease in Latin America has been reduced in recent decades, the United States and a number of non-endemic countries in Europe and the Western Pacific Region have experienced a considerable increase in the number of *T. cruzi-*infected individuals. By the 1980s there had still been no official estimate of the number of *T. cruzi-*infected individuals in the United States disseminated by the CDC or WHO, and only a small number of cases had been reported in Europe. Currently, the best estimates available place the number of *T. cruzi*-infected individuals in the United States at over 300,000 [14, 28], with an additional 80,000 residing in Europe [51, 88], and over 10,000 in other non-endemic countries, most notably Australia, Canada, and Japan [71, 89]. These numbers may even be understating the extent of the Chagas disease burden in countries outside of Latin America which lack universal screening systems and whose physicians are often poorly trained in recognizing the disease. Also, the majority of infected individuals in non-endemic countries are Latin American immigrants who often have disproportionately poor access to health care, and are difficult to accurately track and assess from an epidemiological standpoint.

By any estimate, Chagas disease remains a major public health burden today. To illustrate its seriousness, Chagas disease has been labeled “The New HIV/AIDS of the Americas” by prominent scientists due to a number of similarities in epidemiology and societal impact between the two diseases [49]. Chagas disease and AIDS are both chronic conditions caused by blood-borne pathogens that require expensive long-term treatment, and for which there is no effective cure or preventive vaccine. Both diseases affect large numbers of people and exact a substantial social and economic toll. Currently, the number of people infected with *T. cruzi* in Central and South America is estimated to be over five times the number of people infected with HIV in the same region; however, the global number of HIV infections is higher than the number of *T. cruzi*-infected individuals [71]. Both diseases pose infection risks to recipients of blood transfusion and organ donation and to children of untreated infected mothers. Moreover, both diseases are highly stigmatized and disproportionately affect individuals living in poverty and least able to access the medical and social support necessary for maintaining the highest possible quality of life. These striking comparisons may prove to be effective at increasing awareness of the seriousness of Chagas disease; however, substantial differences between the infectivity, mortality rate and treatment of the two diseases impose considerable limitations on their comparison. Whereas AIDS is almost always fatal and control of HIV infection requires lifelong antiretroviral treatment, only 20–30% of people infected with Chagas disease will develop potentially fatal cardiomyopathy in their lifetime, and *T. cruzi* infection is relatively controllable with short-term treatment compared with HIV.

One of the most noteworthy recent developments in broadening understanding of Chagas disease regards not what occurs during *T. cruzi* infection, but rather where *T. cruzi* infection occurs. In addition to acknowledging the rapidly increasing number of *T. cruzi*-infected individuals currently residing in non-endemic countries, it is important to note that not all cases of *T. cruzi* infection that occur outside of Latin America involve Latin American immigrants who were infected in their countries of origin. One cause of newly acquired *T. cruzi* infections in countries such as the United States, Spain, Switzerland, and, most recently, Japan is congenital transmission [24, 50, 51, 73]. Although the precise number of congenital Chagas cases in non-endemic countries is unknown [21], it is estimated that 40,000 pregnant women and 2000 newborns are infected with *T. cruzi* in North America (Canada, Mexico, and the United States) alone. Because *T. cruzi*-infected neonates are often asymptomatic or exhibit non-specific clinical signs, and obstetrician-gynecologists in non-endemic countries often have limited awareness of Chagas disease and may be less likely to provide the prompt diagnosis and treatment that is crucial for preventing disease progression, congenital transmission is a serious concern that warrants increased attention [24]. Adoption of potentially infected children from endemic regions as well as travel to endemic regions by foreigners may also result in a number of cases of *T. cruzi* infection in non-endemic countries [28].

There are several additional ways that individuals living outside of Latin America may acquire *T. cruzi* infection without having lived in or being born to a mother from an endemic region: receipt of contaminated blood products or organs, vectorial transmission, and laboratory accidents. Of these, transmission through blood transfusion or organ transplantation has resulted in the highest number of *T. cruzi* infections in non-endemic countries, with approximately 20 infections having been recorded in Canada, Spain, and the United States; most or all of these involving donors originating from endemic countries [11, 13, 88]. The United States and France did not begin screening blood donors for the presence of *T. cruzi* until 2007, and many non-endemic countries either did not start screening for the parasite until even more recently, or still do not engage in widespread screening. As of 2014, Japan still has not implemented routine laboratory-based screening of donated blood for the presence of *T. cruzi*, and the country also does not customarily screen pregnant mothers for *T. cruzi* infection, relying instead on a questionnaire to determine whether self-reported risk factors warrant individualized testing [50]. As of January 2014, at least 1900 seropositive donors have been reported in the United States since testing began in 2007, according to the American Association of Blood Banks website. The proportion of US blood donors testing positive for *T. cruzi* is highest in areas with large numbers of Latin American immigrants, such as Los Angeles and Miami, where the seropositive rates have been reported as 1 in 7500 and 1 in 9000, respectively [56].

As of 2011, only 7% of the 58 organ procurement organizations active in the United States routinely screened all organ donors serologically for *T. cruzi*, with an additional 12% employing selective screening of high-risk donors [79]. In 2008, 17 donor organs being considered for transplantation into recipients were discarded following a positive test for *T. cruzi* [79]. Because *T. cruzi-*infected individuals may remain asymptomatic for decades before developing life-threatening health problems, it is conceivable that additional people have already been infected with the parasite following the receipt of a blood or organ donation and are unaware of their infection status.

Of the 65 cases of *T. cruzi* infection known to have been acquired via laboratory accidents, at least 11 occurred in the United States or Europe [46]. Although not a similar threat outside of North America, at least seven cases of vectorial transmission of *T. cruzi* have been verified in the United States since 1955 [13, 22, 35, 47, 65, 76, 90]. The range of triatomine bugs capable of transmitting *T. cruzi* extends across twenty-six states in the southern half of the country, though infestation of domestic dwellings by triatomine bugs is rare and usually only occurs under atypical conditions, such as following severe droughts [10, 13, 72, 75].

Oral transmission of *T. cruzi*, which usually occurs due to ingestion of fresh sugar cane or açaí berry juice made from plants harboring infected triatomine bugs, is now the primary cause of *T. cruzi* infection in some areas of Latin America, such as the Amazonian region of Brazil [80]. Oral transmission has resulted in over 1000 cases of acute Chagas disease in Latin America since the year 2000 [80]. This mode of transmission is considered an emerging threat because outbreaks are sporadic, difficult to predict, and have shown no signs of declining in frequency or severity. The main threat that this route poses to individuals in non-endemic countries is the risk to tourists traveling to areas where consumption of contaminated beverages or foods is most likely.

## The future of Chagas disease

Development of an effective vaccine or other new and improved therapies is a crucial, and perhaps the most anticipated and important next step in the fight against Chagas disease [53, 55, 68]. A number of groups are currently in the advanced stages of developing novel therapies, including both DNA- and antigen-based vaccines [36, 37, 43, 62, 68, 87, 91] as well as other anti-trypanosomal drugs including chemical agents that competitively inhibit the function of critical *T. cruzi* enzymes [16]. Preliminary data demonstrating the efficacy of several of these candidate drugs has been promising, and progress into clinical trials for at least one candidate vaccine which targets two *T. cruzi* antigens (Tc24 and TSA-1) and includes a TLR4 agonist is likely to occur within the next five years [37]. Benefits of a potential vaccine for *T. cruzi* compared with the use of standard chemotherapeutic agents include reduced toxicity, allowing for expanded use in chronic patients and patients with comorbidities, potential use during pregnancy to prevent congenital transmission, increased protection against cardiac complications, and removal of treatment barriers associated with the effort and cost of administering repeated drug treatments. Due to the controversial proposition that *T. cruzi*-induced autoimmunity may play a role in the cardiac pathogenesis of human Chagas disease, it is recommended, even by experts who agree that protective anti-*T. cruzi* immunity can be promoted by vaccines without the risk of eliciting pathogenic autoimmunity, that vaccine candidates continue to be monitored and tested for this potential risk [36]. In the absence of a rapidly effective cure, work must continue on the development of improved treatments and prognostic indicators for sustained organ damage in chronic Chagas patients, including expanding insight into genetic factors that may influence susceptibility to disease progression and reparative stem cell therapies to ameliorate cardiac damage [8, 29, 38].

Continued progress toward limiting the spread of Chagas disease also requires sustained efforts at widespread vector control [60], refinement of infection and disease risk assessment [38, 64], improvement in the quality of diagnostic tests for screening individuals and the blood supply [1], increased availability of existing therapies and diagnostic tests [57], and expansion of surveillance programs in endemic regions [27, 60]. The Amazonian region of Brazil is of particular importance for surveillance efforts due to the high rate of habitat conversion of previously uninhabited wilderness into dwellings and farmland, creating potential points of contact between *T. cruzi* and humans [27, 60]. Also, oral transmission of *T. cruzi* is more prevalent here than anywhere else in the world, and will likely continue to cause a significant number of new infections for many years to come [78, 80]. In addition to maintaining and expanding the aforementioned efforts, endemic countries must continue providing care for the approximately 10 million people already infected with *T. cruzi*, which will include hospitalizations and other long-term and expensive treatments for many thousands of individuals.

Another important concern in the ongoing effort to control Chagas disease is maintaining a high enough level of political priority to promote and fund surveillance and research at the levels necessary to prevent lapses or regression in the success of public health programs [27, 45, 60]. As stated by Dias et al. [33], “the greatest risk to the current successful trend in Chagas disease control comes, in a sense, from the success that has been achieved.” To elaborate, knowledge of the widespread reduction in the prevalence and risk of *T. cruzi* infection that has already been achieved may, over time, result in a loss of interest in and commitment to providing and improving surveillance and research and treatment strategies, or a large-scale shifting of resources to more emergent issues. Development of complacency or a lackadaisical attitude toward Chagas disease would carry the risk of allowing a progressive reestablishment of *T. cruzi* transmission and losing the ability to effectively deal with future outbreaks. Therefore, it is important to maintain robust, centralized public health programs to track and treat *T. cruzi* transmission, as well as continue to educate the public about the risks of and preventive strategies for the disease.

Additionally, efforts to control the spread of Chagas disease must include increased emphasis on monitoring and controlling globalization of the disease, including the emerging threat of Chagas disease in Europe, Japan, Australia, and the United States and the need for increased surveillance in those areas [6, 9, 13, 27, 28, 39, 49, 60]. Unlike in most regions of Latin America, the number of *T. cruzi-*infected individuals is rising considerably in non-endemic countries such as the United States, and over 10% of the global healthcare burden related to Chagas disease already originates outside Latin America [54]. Also unlike endemic countries, health care professionals and the public in historically non-endemic countries lack a long history of training and awareness in how to prevent, detect, and treat the disease. In areas where Chagas disease is newly emerging as a major public health concern, as in endemic countries, a crucial part of any successful campaign to limit the spread of *T. cruzi* infection is education. It is recommended that training in practical methods for identifying and protecting against the vector and for recognizing and seeking treatment for symptoms of potential infection be widely disseminated to primary school educators and community health advocates, in addition to physicians and other health care providers [25]. Increasing awareness of Chagas disease among children is also important since many infections occur during childhood, and because it is the youngest generation that will become the future researchers, health care providers and public policy-makers tasked with the challenge of breaking the self-propagating cycle of a disease that disproportionately affects the poor and further contributes to poverty due to loss of productivity and healthcare costs [17, 25, 78].

## Conclusion

There is a growing consensus that Chagas disease, no longer confined to poor rural areas of Latin America, is now a worldwide public health concern and will remain so for the foreseeable future. However, after decades of improvements in surveillance, treatment, and vector-eradication strategies, effective elimination of the disease in the near future is becoming an increasingly attainable goal. Future success in the fight against Chagas disease is dependent upon effective management of newly emerging infectious foci, maintenance of high levels of public awareness and government interest in controlling the disease, and continued improvements in diagnostic, therapeutic, and surveillance tools. Lessons learned from the past 100 years of combating *T. cruzi* infection must continually be applied and improved upon in order for the next 100 years to yield continued progress against and possibly even eradication of Chagas disease.

## Conflict of interest

The author declares no conflicts of interest.
